# Supplementary material for: Large-scale whole-exome sequencing analyses identified protein-coding variants associated with immune-mediated diseases in 350,770 adults
Source: Nat Commun. 2024 Jul 15;15:5924. doi: 10.1038/s41467-024-49782-0 (PMC11250857; doi:10.1038/s41467-024-49782-0)
Supplement: Supplementary file 1 — Supplementary Information [file 41467_2024_49782_MOESM1_ESM.pdf]

Supplementary Information for

Large-scale whole exome sequencing analyses identified protein-coding variants associated with immune-mediated diseases in 350770 adults

Content

Supplementary Figures 2

Supplementary Fig.1 Q-Q plots for exome-wide rare variant analysis 2

Supplementary Fig.2 Pleiotropic effects on clinical outcomes 11

Supplementary Fig.3 Longitudinal IMDs risk for putatively pathogenic variations 12

Supplementary Fig.4 Proteomic-wide analysis between identified genes and 1464 proteins 14

Supplementary Fig.5 The PPI network of *PSMB9* on IL10 levels 16

Supplementary Fig.6 PPI analysis of 164 genes (a) and their disease-associated pathway clusters (b) 17

Supplementary Fig.7 Specific cell type analysis using scRNA-seq data 19

Supplementary Methods 20

Supplementary Method 1 Quality control of exome sequencing 20

Supplementary Method 2 Relationship inference, kinship matrix, and principal component analysis 22

Supplementary Method 3 Genotype and imputation of GWAS 24

Supplementary Figures

Supplementary Fig.1 Q-Q plots for exome-wide rare variant analysis

a

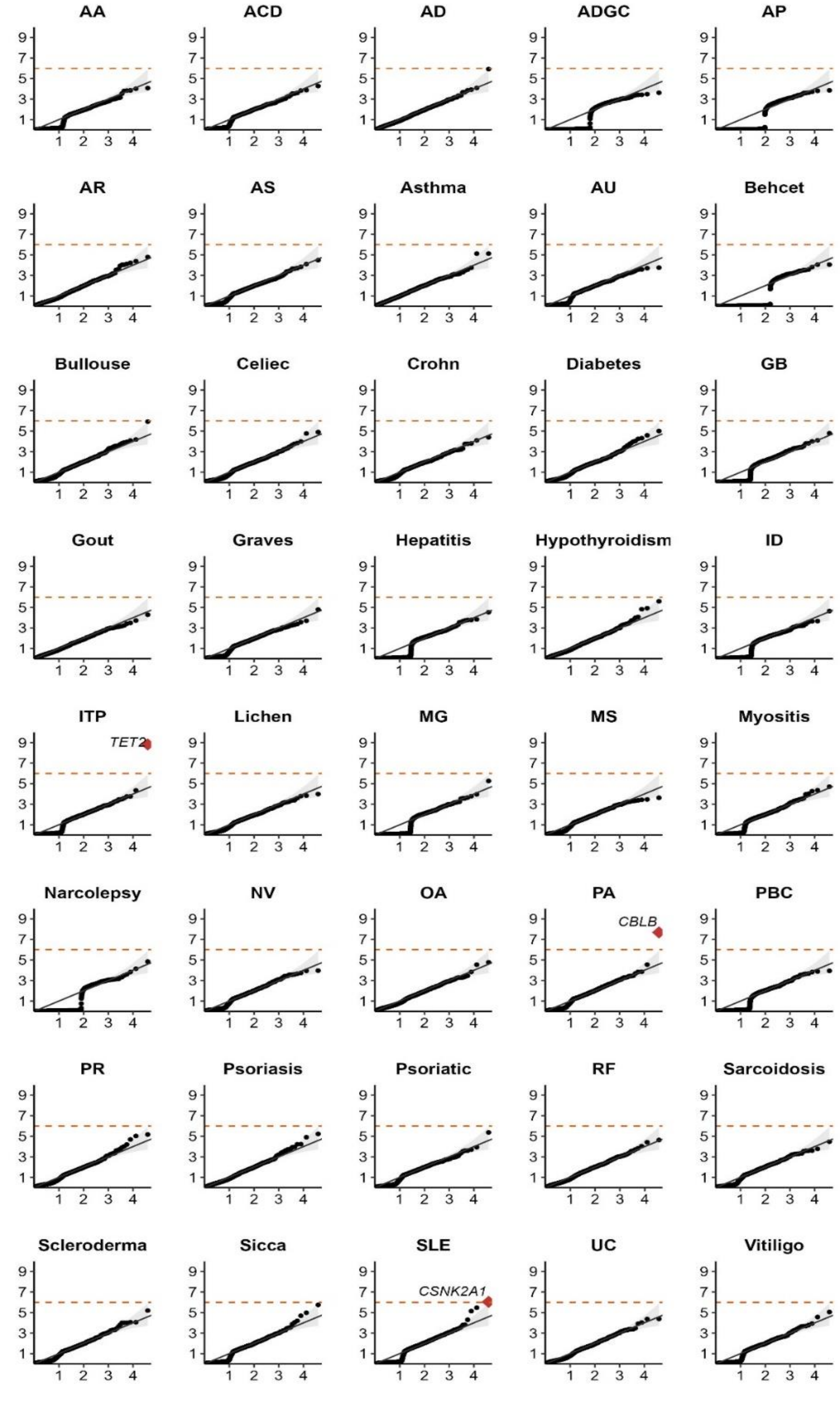

b

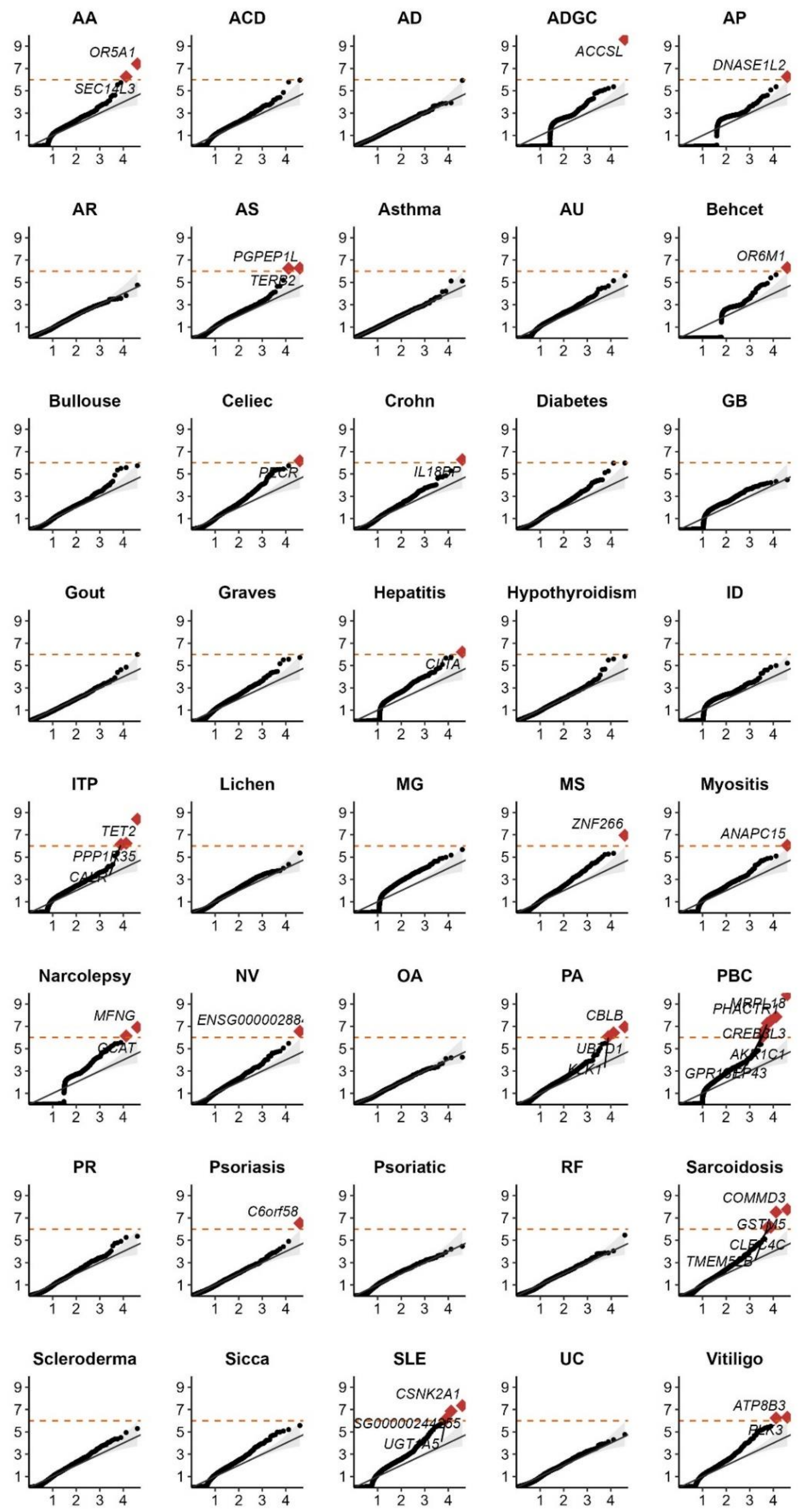
AA
OR5A1
ACD
AD
ADGC
ACCSL
AP
DNASE1L2
AR
AS
PGPEP1L
Asthma
AU
Behcet
OR6M1
Bullouse
Celiec
Crohn
Diabetes
GB
Gout
Graves
Hepatitis
Hypothyroidism
ID
ITP
TET2
Lichen
MG
MS
ZNF266
Myositis
ANAPC15
Narcolepsy
MFNG
NV
OA
PA
CBLB
PBC
PR
Psoriasis
C6orf58
Psoriatic
RF
Sarcoidosis
COMMD3
Scleroderma
Sicca
SLE
CSNK2A1
UC
Vitiligo
ATP8B3

c

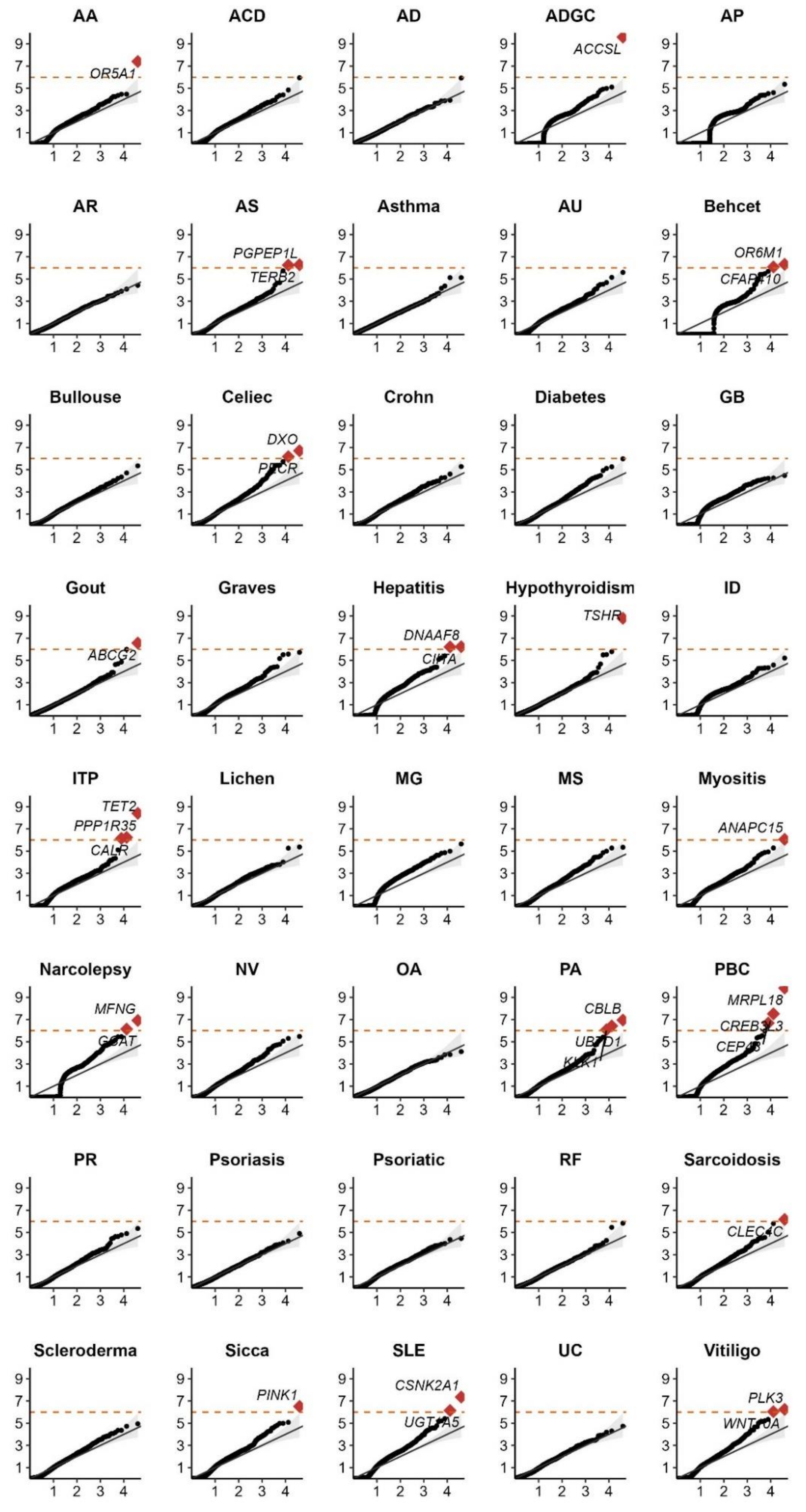
AA
OR5A1
ACD
AD
ADGC
ACCSL
AP
AR
AS
PGPEP1L
TERB2
Asthma
AU
Behcet
OR6M1
Bullouse
Celiec
DXO
Crohn
Diabetes
GB
Gout
ABCG2
Graves
Hepatitis
DNAAF8
Hypothyroidism
TSHR
ID
ITP
TET2
PPP1R35
Lichen
MG
MS
Myositis
ANAPC15
Narcolepsy
MFNG
NV
OA
PA
CBLB
PBC
MRPL18
PR
Psoriasis
Psoriatic
RF
Sarcoidosis
Scleroderma
Sicca
PINK1
SLE
CSNK2A1
UC
Vitiligo
PLK3

d

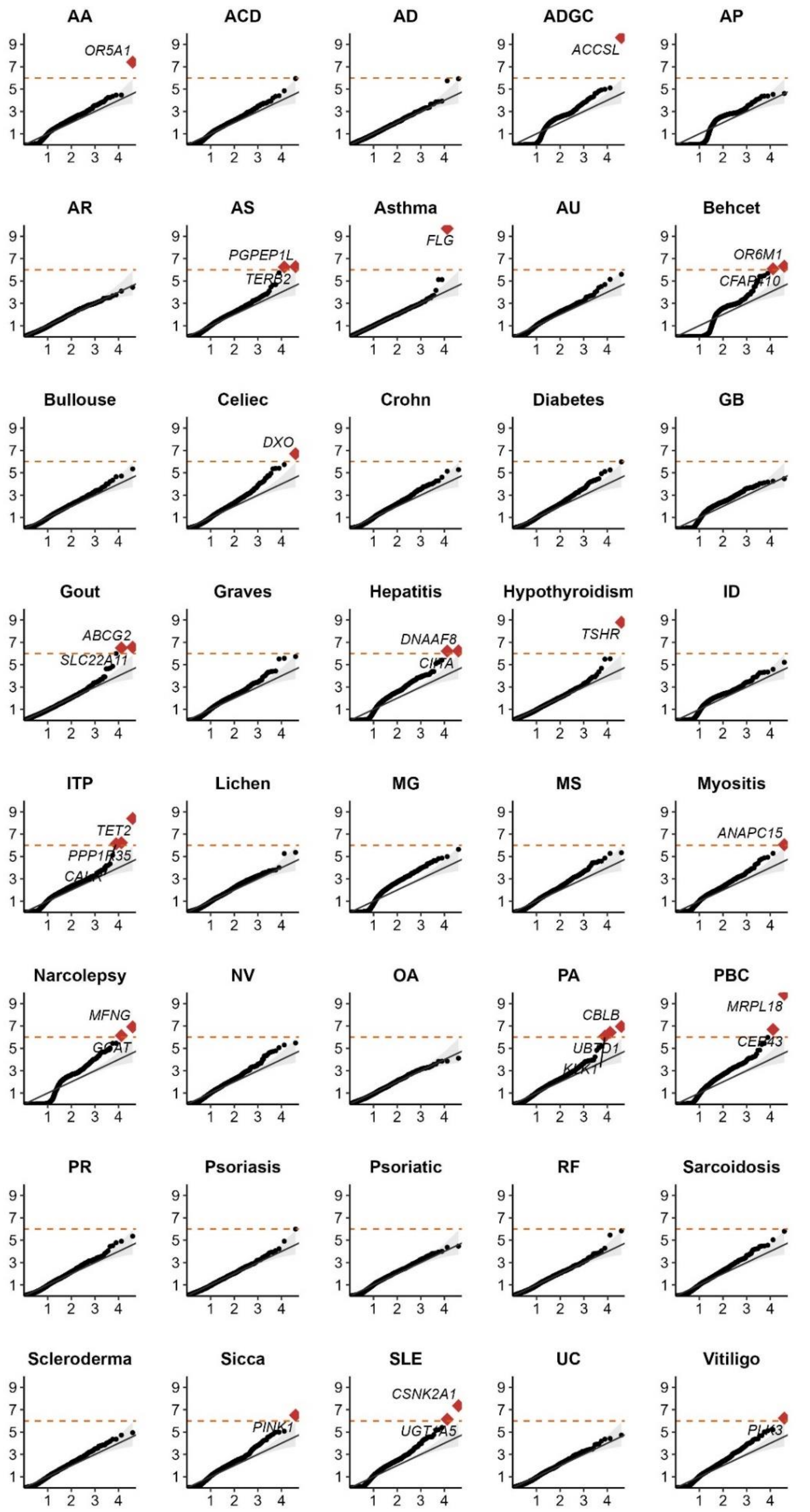
AA
OR5A1
ACD
AD
ADGC
ACCSL
AP
AR
AS
PGPEP1L
Asthma
FLG
AU
Behcet
OR6M1
Bullouse
Celiec
DXO
Crohn
Diabetes
GB
Gout
ABCG2
Graves
Hepatitis
DNAAF8
Hypothyroidism
TSHR
ID
ITP
TET2
Lichen
MG
MS
Myositis
ANAPC15
Narcolepsy
MFNG
NV
OA
PA
CBLB
PBC
MRPL18
PR
Psoriasis
Psoriatic
RF
Sarcoidosis
Scleroderma
Sicca
PINK1
SLE
CSNK2A1
UC
Vitiligo

e

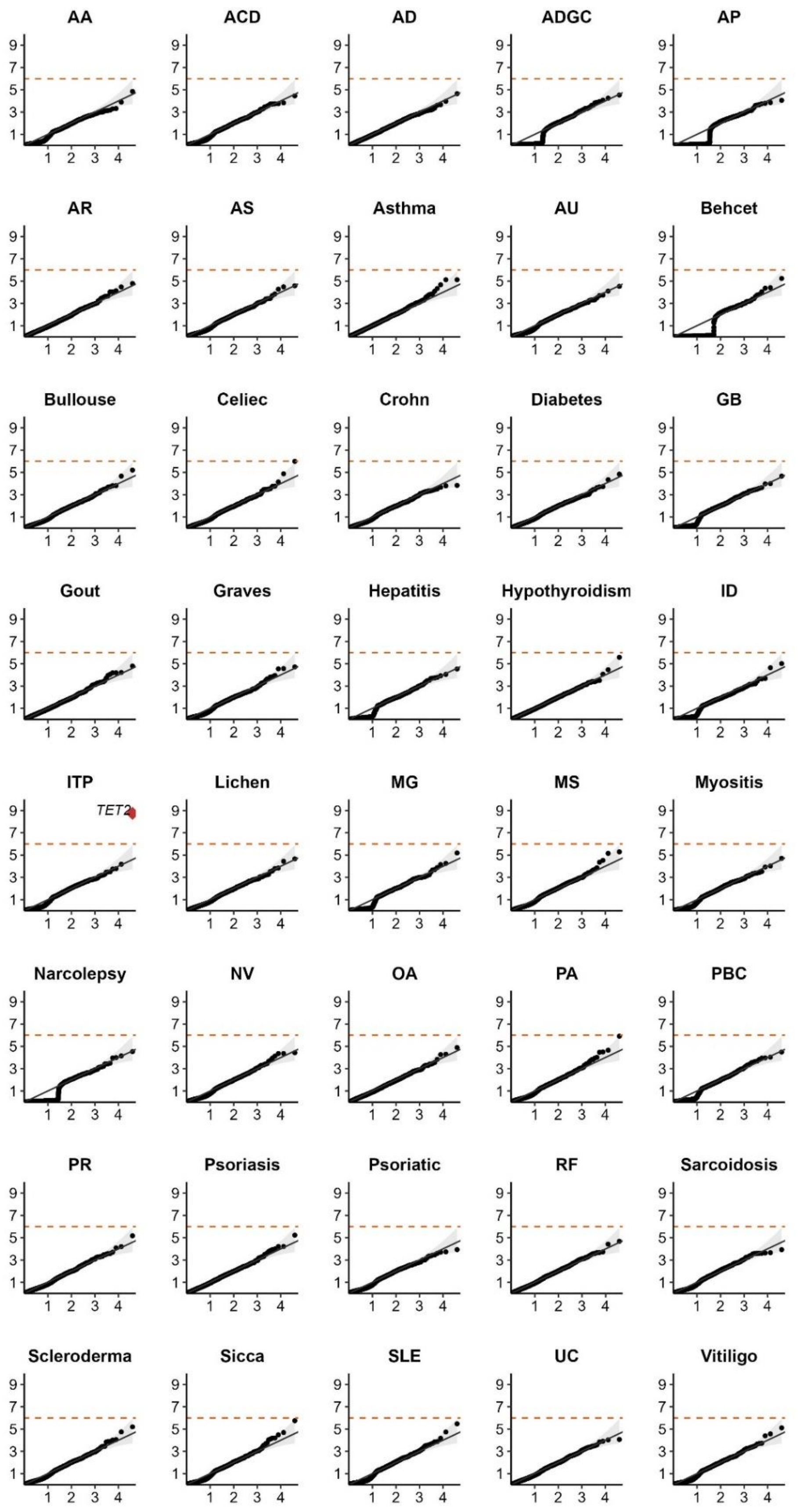
AA
ACD
AD
ADGC
AP
AR
AS
Asthma
AU
Behcet
Bullouse
Celiec
Crohn
Diabetes
GB
Gout
Graves
Hepatitis
Hypothyroidism
ID
ITP
TET2
Lichen
MG
MS
Myositis
Narcolepsy
NV
OA
PA
PBC
PR
Psoriasis
Psoriatic
RF
Sarcoidosis
Scleroderma
Sicca
SLE
UC
Vitiligo

f

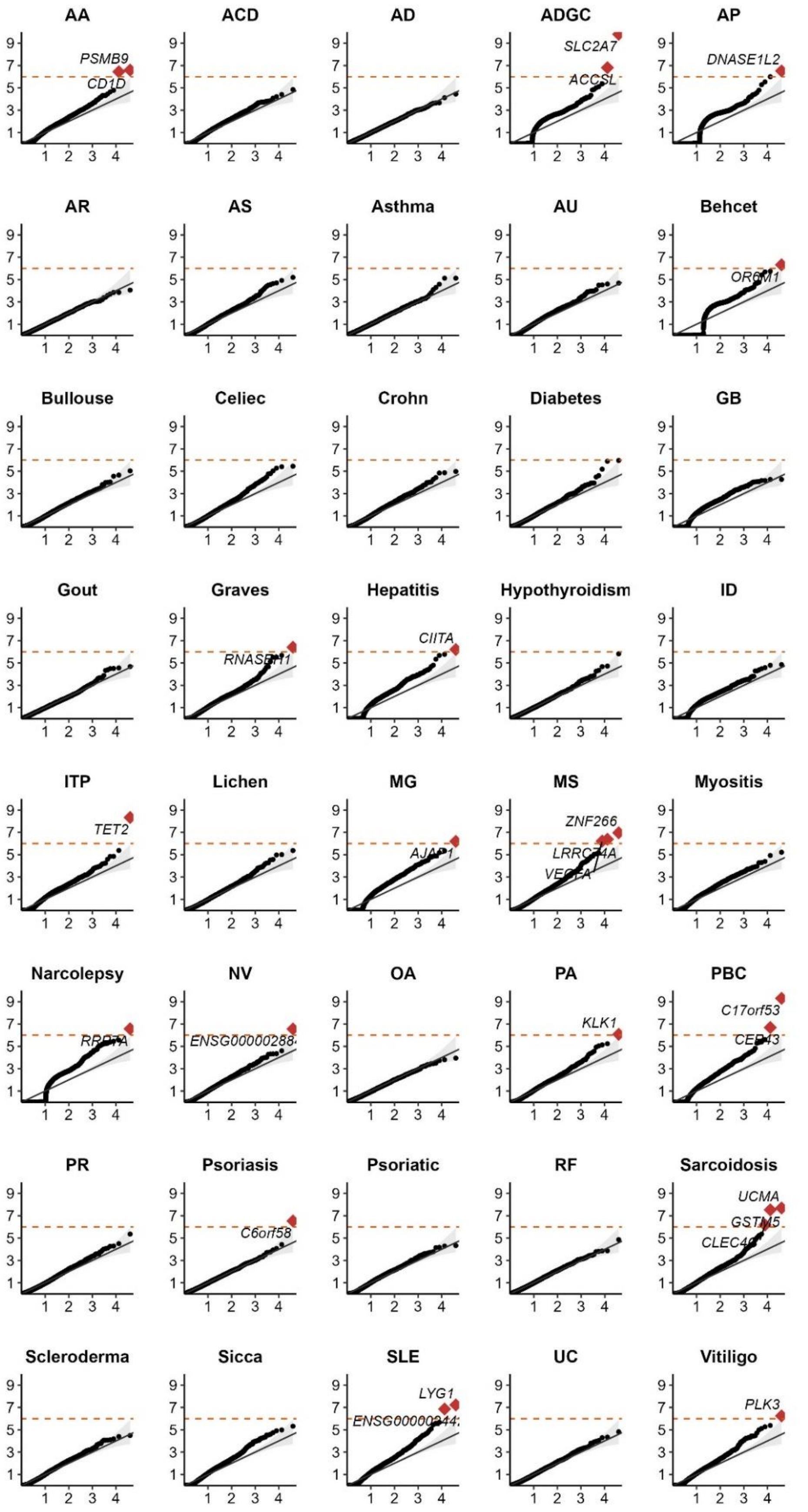
AA
PSMB9
CD1D
ACD
AD
ADGC
SLC2A7
ACCSL
AP
DNASE1L2
AR
AS
Asthma
AU
Behcet
OR6M1
Bullouse
Celiec
Crohn
Diabetes
GB
Gout
Graves
RNASEH1
Hepatitis
CIITA
Hypothyroidism
ID
ITP
TET2
Lichen
MG
AJAP1
MS
ZNF266
LRRC74A
VEGFA
Myositis
Narcolepsy
RRP7A
NV
OA
PA
KLK1
PBC
C17orf53
CEP43
PR
Psoriasis
C6orf58
Psoriatic
RF
Sarcoidosis
UCMA
GSTM5
CLEC4C
Scleroderma
Sicca
SLE
LYG1
UC
Vitiligo
PLK3

g

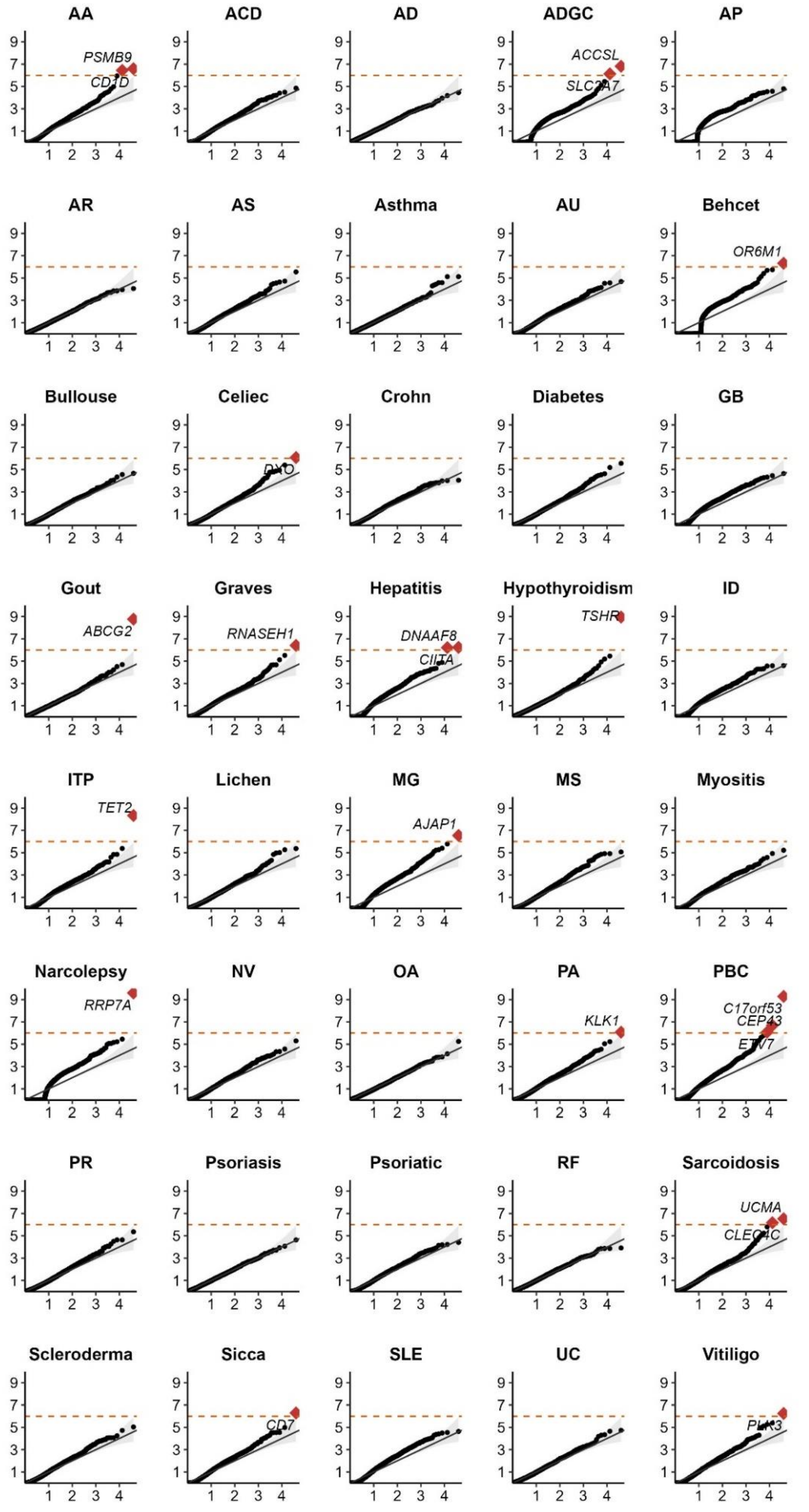

AA
PSMB9
CD1D
ACD
AD
ADGC
ACCSL
SLC7A7
AP
AR
AS
Asthma
AU
Behcet
OR6M1
Bullouse
Celiec
DAO
Crohn
Diabetes
GB
Gout
ABCG2
Graves
RNASEH1
Hepatitis
DNAAF8
CIITA
Hypothyroidism
TSHR
ID
ITP
TET2
Lichen
MG
AJAP1
MS
Myositis
Narcolepsy
RRP7A
NV
OA
PA
KLK1
PBC
C17orf53
CEP43
ETV7
PR
Psoriasis
Psoriatic
RF
Sarcoidosis
UCMA
CLEC4C
Scleroderma
Sicca
CD7
SLE
UC
Vitiligo
PLK3

h

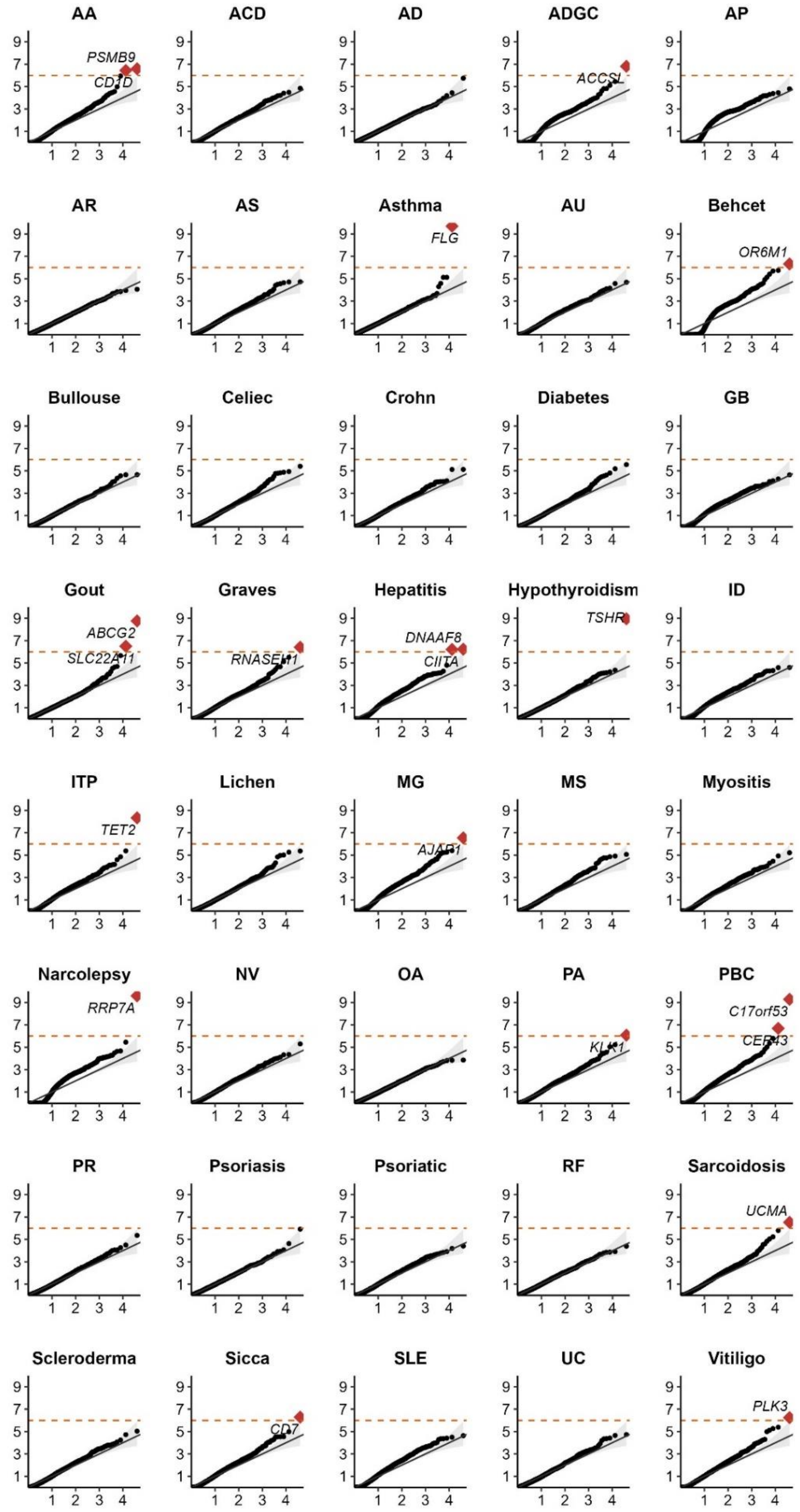
AA
PSMB9
CD1D
ACD
AD
ADGC
ACCSL
AP
AR
AS
Asthma
FLG
AU
Behcet
OR6M1
Bullouse
Celiec
Crohn
Diabetes
GB
Gout
ABCG2
SLC22A11
Graves
RNASEH1
Hepatitis
DNAAF8
CIITA
Hypothyroidism
TSHR
ID
ITP
TET2
Lichen
MG
AJAP1
MS
Myositis
Narcolepsy
RRP7A
NV
OA
PA
KLK1
PBC
C17orf53
CEP43
PR
Psoriasis
Psoriatic
RF
Sarcoidosis
UCMA
Scleroderma
Sicca
CD7
SLE
UC
Vitiligo
PLK3

Q-Q plots of results from exome-wide rare variant analysis. The *y*-axis represents the observed-$\log_{10}(P)$, while the *x*-axis represents the expected under the null-hypothesis. *P* values shown are two-sided. The red dotted horizontal line represents a threshold of $P=1\times10^{-6}$. Panel a to d for exome-wide rare analysis of lof variants, with MAF from <1e-5 (a),1e-5~1e-4 (b),1e-4~1e-3 (c),1e-3~0.01 (d); panel e to h for exome-wide rare analysis of lof+missense variants, with MAF from <1e-5 (e),1e-5~1e-4 (f),1e-4~1e-3 (g),1e-3~0.01 (h).

Abbreviations: AA, alopecia areata; ACD, allergic contact dermatitis; AD, atopic dermatitis; ADGC, allergic and dietetic gastro-enteritis and colitis; AP, allergic purpura; AR, allergic rhinitis; AS, ankylosing spondylitis; AU, allergic urticaria; Behcet, Behcet's disease; Bullouse, Bullouse disorders; Coeliac, coeliac disease; Crohn, Crohn's disease; Diabetes, diabetes mellitus (Type I); GB, Guillain-Barre syndrome; Graves, Graves' disease; Hepatitis, autoimmune hepatitis; Hypothyroidism, autoimmune hypothyroidism; ID, immunodeficiency with predominantly antibody defects; ITP, idiopathic thrombocytopenic purpura; Lichen, Lichen planus; MG, myasthenia gravis; MS, multiple sclerosis; NV, necrotizing vasculopathies; OA, osteoarthritis; PA, pernicious anaemia; PBC, primary biliary cirrhosis; PR, polymyalgia rheumatica; RF, rheumatic fever; Sicca, Sicca syndrome (Sjogren's syndrome); SLE, systemic lupus erythematosus; UC, ulcerative colitis.

Supplementary Fig.2 Pleiotropic effects on clinical outcomes

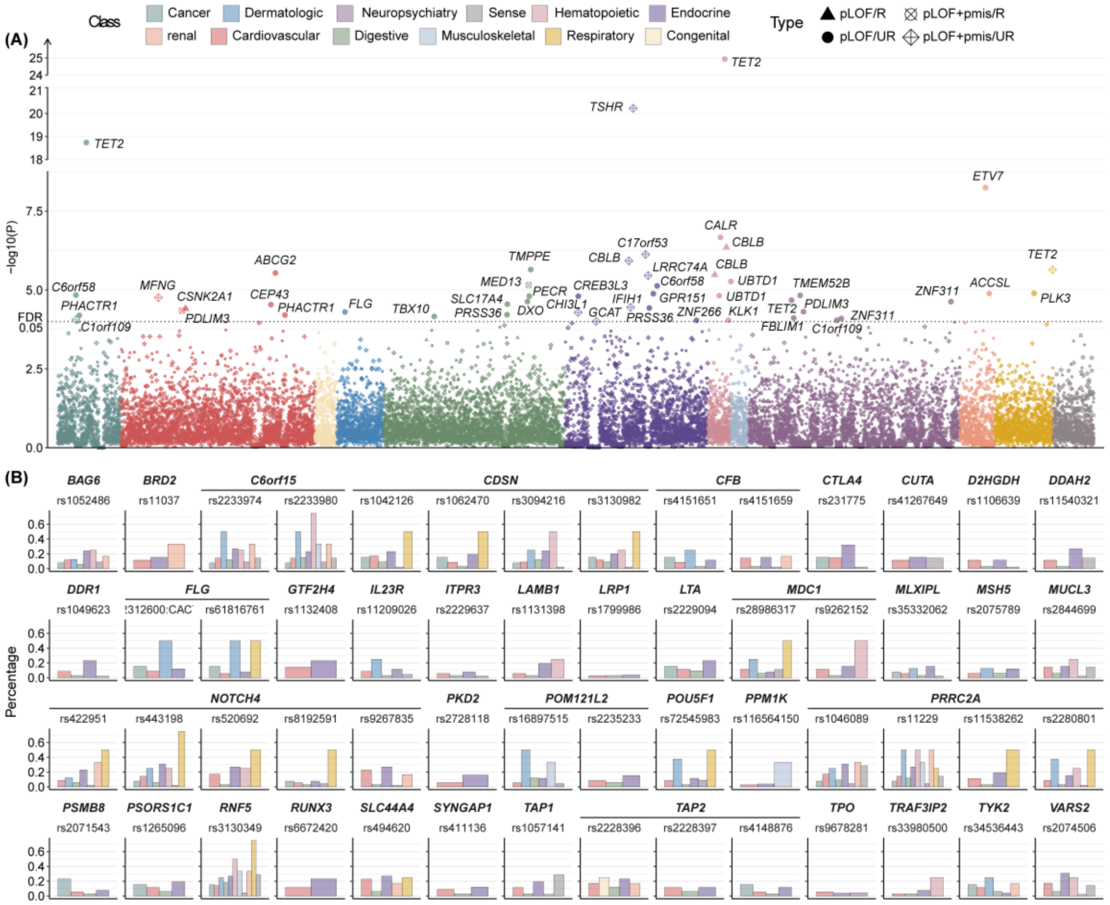

a, Results from gene-based collapsing tests with clinical outcomes for identified rare variants. The x-axis represented the categories of selected conditions and the y-axis represented the -log$_{10}$(P). **b,** Results from single-variant association tests with clinical outcomes for identified common variants. The bar plots displayed number of significant associations of each identified variant with clinical outcomes.

Abbreviations: FDR, false discovery rate; pLOF, predicted loss-of-function; UR, ultra-rare; R, rare; pmis, predicted deleterious missense.

Supplementary Fig.3 Longitudinal IMDs risk for putatively pathogenic variations

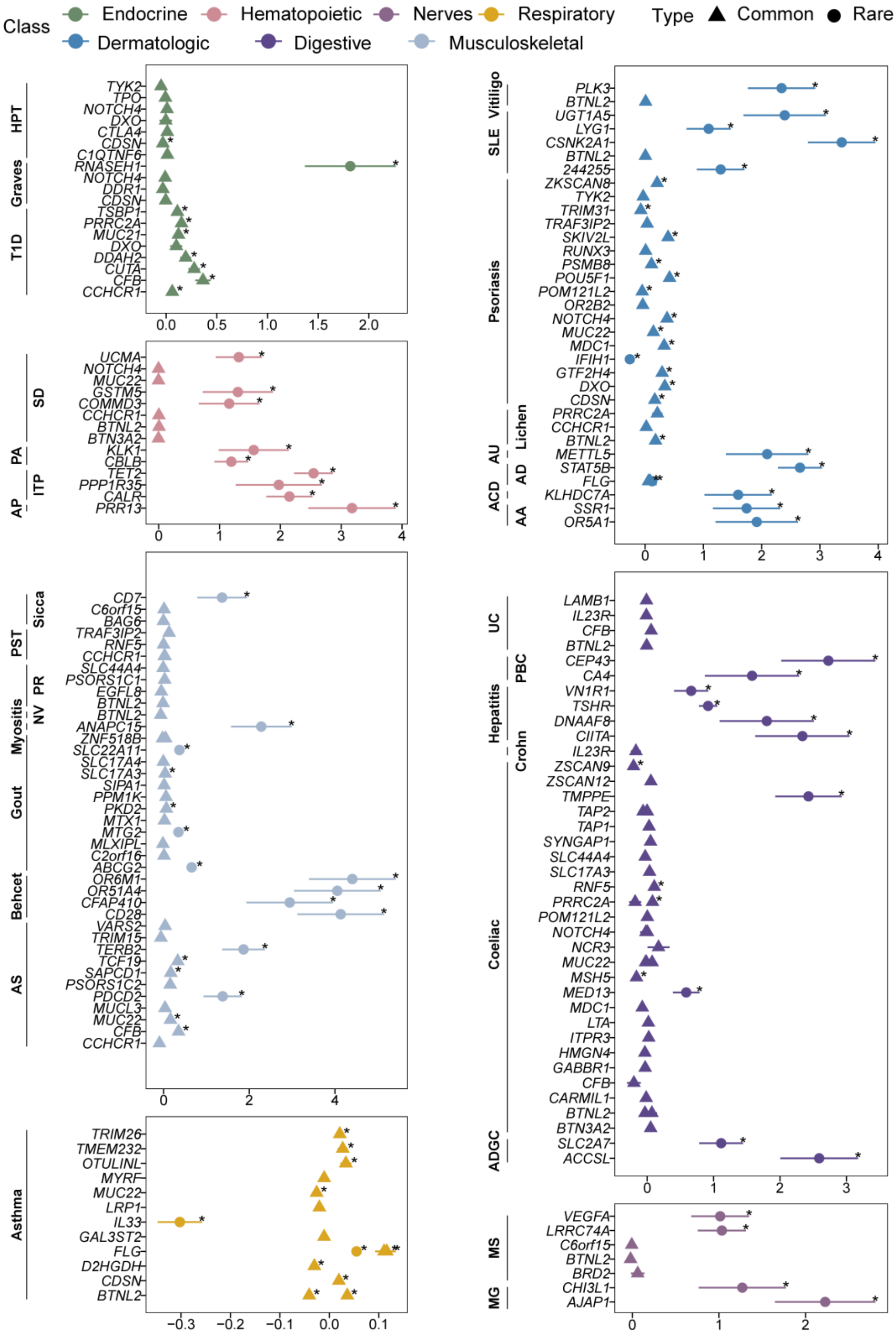

Circles and squares represent hazard ratios for rare or common mutation carriers, and horizontal lines indicate corresponding 95% confidence intervals (CIs) around hazard ratios (HRs). HRs

were calculated using Cox proportional hazards regression analysis after adjustments for sex and the first ten PCs.

Abbreviations: IMD, immune-mediated disease; PC, principal component; HR, hazard ratio; CI, confidence interval; HPT, Autoimmune hypothyroidism; Grave’s, Grave’s disease; T1D, Diabetes mellitus (Type I); SD, Sarcoidosis; PA, Pernicious anaemia; ITP, Idiopathic thrombocytopenic purpura; AP, Allergic purpura; Sicca, Sicca syndrome (Sjogren's syndrome); PST, Psoriatic and enteropathic arthropathies; PR, Polymyalgia rheumatica; NV, Necrotizing vasculopathies; Behcet, Behcet's disease; AS, Ankylosing spondylitis; SLE, Systemic Lupus erythematosus; Lichen, Lichen planus; AU, Allergic urticaria; AD, Atopic Dermatitis; ACD, Allergic contact dermatitis; AA, Alopecia areata; UC, Ulcerative colitis; PBC, Primary biliary cirrhosis; Hepatitis, Autoimmune hepatitis; Crohn, Crohn's disease; Coeliac, Coeliac disease; ADGC, Allergic and dietetic gastro-enteritis and colitis; MS, Multiple sclerosis; MG, Myasthenia gravis.

Supplementary Fig.4 Proteomic-wide analysis between identified genes and 1464 proteins

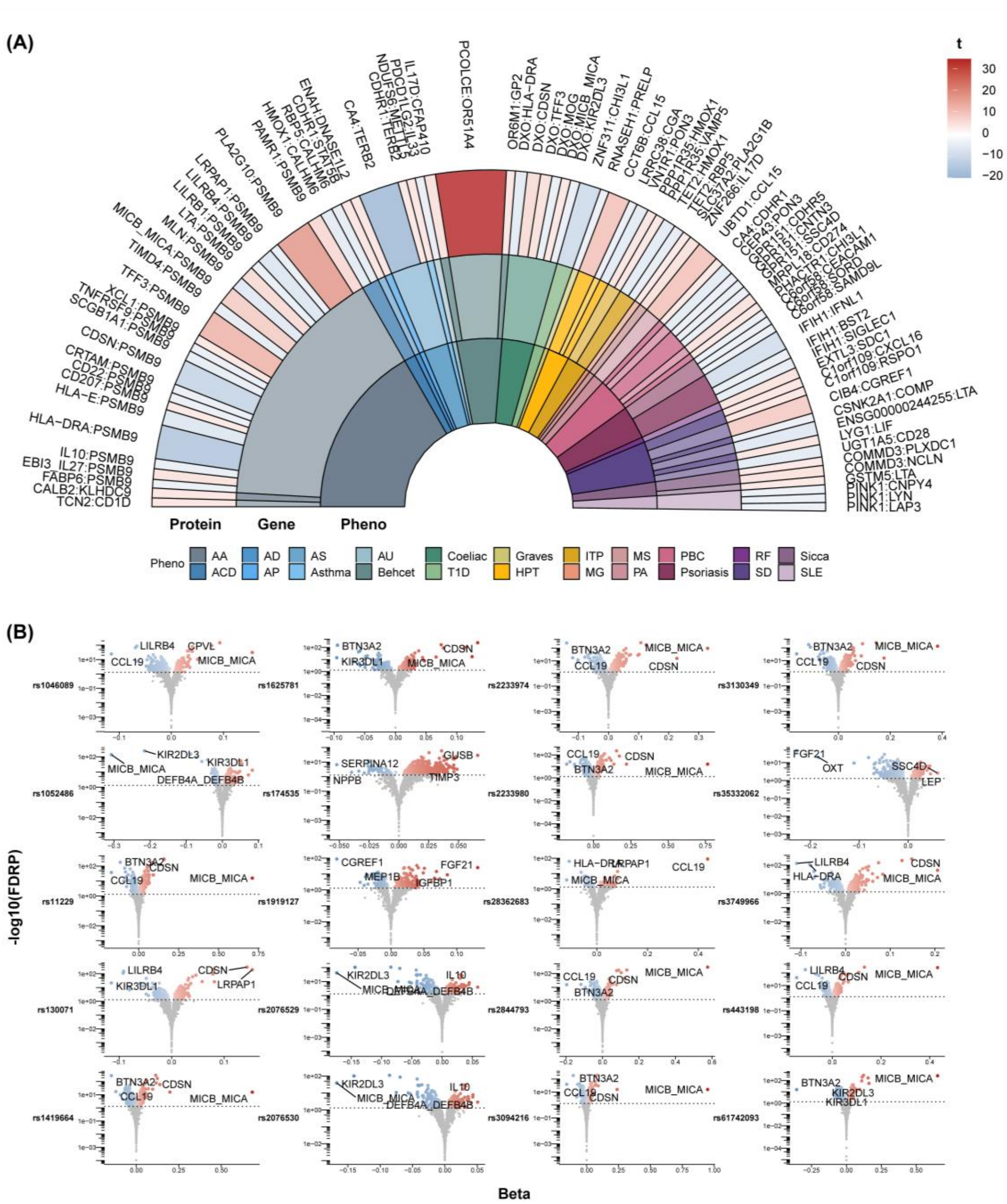

a, A sunburst plot representing significant associations between IMDs (inner circle), their referenced rare genetic variation (middle circle), and modulated protein expressions (outer circle). The color of the outer circle conveys the strength of association measured through coefficients. **b,** Volcano plots of the top 20 genes influencing protein expressions. Blue

indicates downregulation of protein expression, red indicates upregulation, and gray signifies no significant change. For each gene, the top three proteins with the largest coefficients are highlighted.

Abbreviations: IMD, immune-mediated disease; Pheno, Phenotypes; FDR, false discovery rate; AA, Alopecia areata; AD, Atopic Dermatitis; AS, Ankylosing spondylitis; AU, Allergic urticaria; Coeliac, Coeliac disease; Grave’s, Grave’s disease; ITP, Idiopathic thrombocytopenic purpura; MS, Multiple sclerosis; PBC, Primary biliary cirrhosis; Hepatitis, Autoimmune hepatitis; RF, Rheumatic fever; Sicca, Sicca syndrome (Sjogren's syndrome); ACD, Allergic contact dermatitis; AP, Allergic purpura; Behcet, Behcet's disease; T1D, Diabetes mellitus (Type I); HPT, Autoimmune hypothyroidism; MG, Myasthenia gravis; PA, Pernicious anaemia; SLE, Systemic Lupus erythematosus.

Supplementary Fig.5 The PPI network of *PSMB9* on IL10 levels

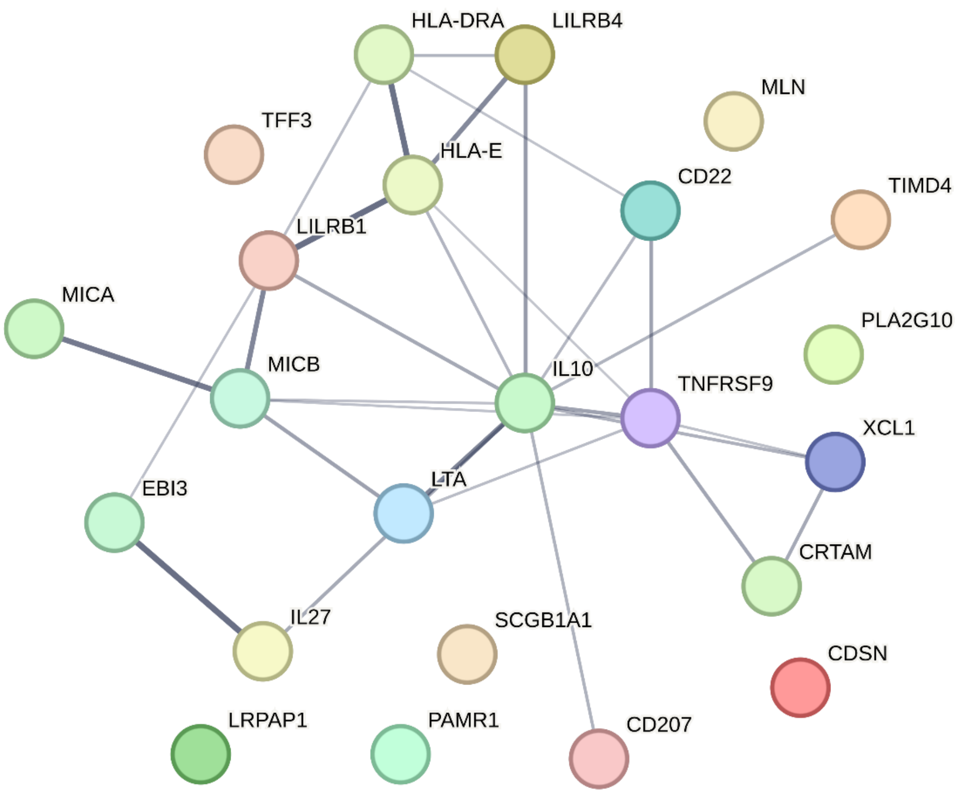

The STRING (Version 10.0, http://string-db.org) was used to predict the relationships among the screened genes. Based on functional and physical protein associations, PPI node pairs with a score of combination > 0.4 (medium confidence) were considered to be significant. A machine learning method (K-means) was utilized to categorize gene clusters. *PSMB9* had 22 PPI significant associations, predominantly with immune-related proteins, especially IL10.

Abbreviations: STRING, Search Tool for the Retrieval of Interacting Genes database; PPI, protein-protein interaction.

Supplementary Fig.6 PPI analysis of 164 genes (a) and their disease-associated pathway clusters (b)

a.

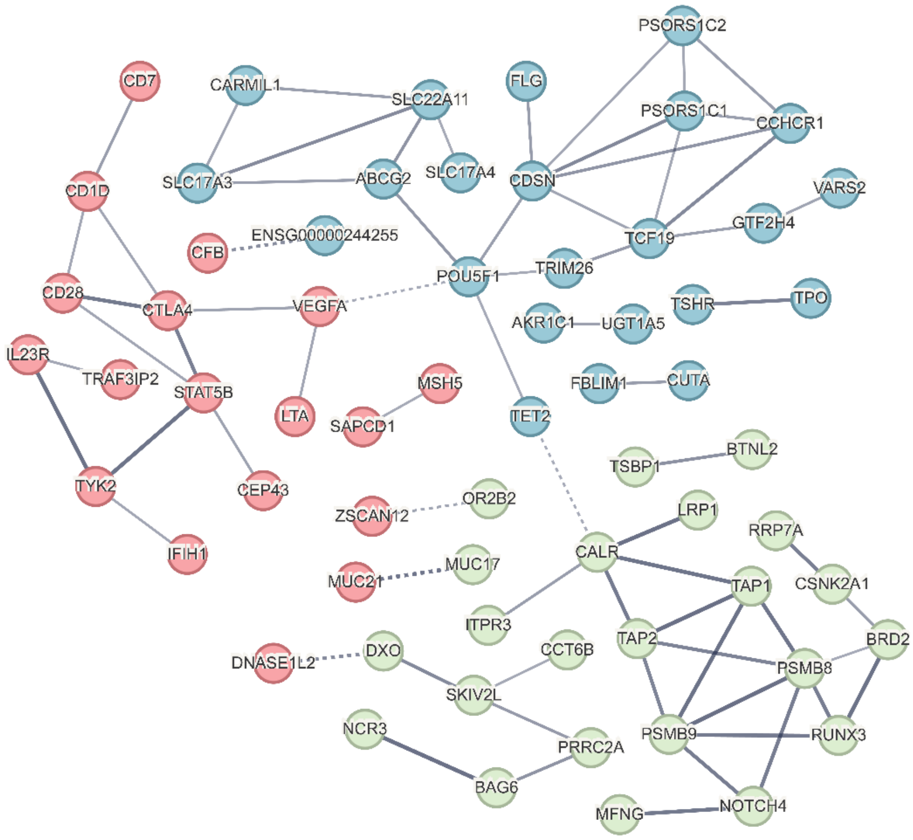

b.

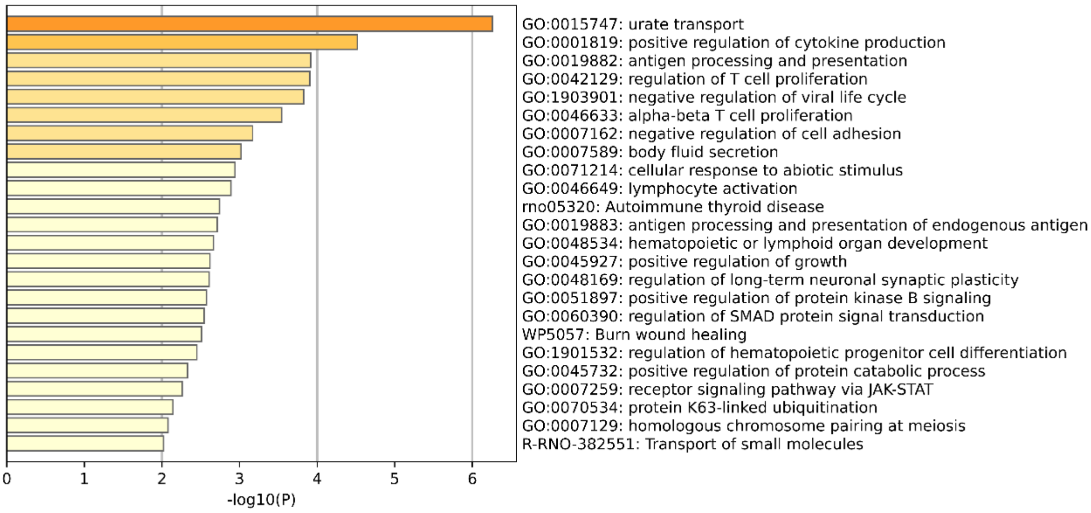

a, STRING was adopted to cluster the PPI network of 164 genes (K-means =3). Orphan genes (i.e., those disconnected in the network) was hidden. The thickness of the lines indicates the

strength of data supporting interactions. The dotted lines indicate the margin of clusters. **b**, MAGMA within FUMA platform was employed to probe three of the biological annotation and pathway compendiums of identified genes (only 161 recognized by FUMA). The top ten significantly enriched pathways of the total gene sets were displayed. FDR adjusted *P*<0.05 was statistically significant.

Abbreviations: FUMA, Functional Mapping and Annotation of Genetic Associations; MAGMA, Multi-marker Analysis of GenoMic Annotation; PPI, protein-protein interaction.

Supplementary Fig.7 Specific cell type analysis using scRNA-seq data

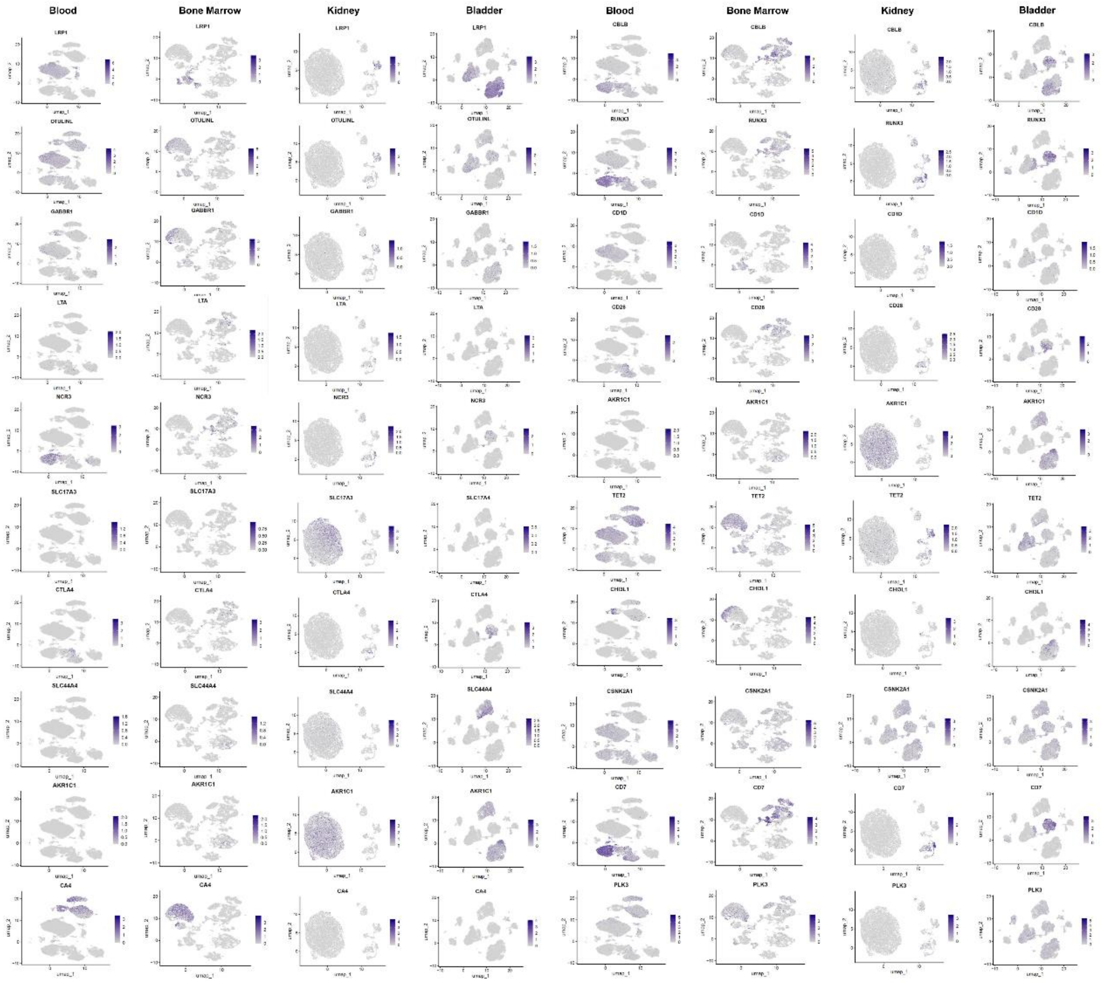

The feature plots of UMAP showed the expression level of genes in different cell types within blood, bone marrow, kidney and bladder.

Abbreviations: UMAP, Uniform Manifold Approximation and Projection; scRNA-seq, single-cell RNA sequencing.

Supplementary Methods

Supplementary Method 1 Quality control of exome sequencing

In addition to the quality control that was performed centrally, we applied extensive genotype-level, variant-level, and sample-level quality control procedures, similar to the previous study(Jurgens et al., 2022), to ensure a high-quality dataset for analyses. To this end, we utilized the exome OQFE data (UKB Field ID 23157, pVCF format) provided by the UK Biobank, which contained calls for about 470,000 sequenced samples.

Genotype quality control

We applied genotype refinement to the raw genotype calls in the pVCF files using Hail. We first split multi-allelic sites to represent separate bi-allelic sites. All calls that did not pass the following filters were set to no-call:

- For homozygous reference calls: Genotype Quality < 20; Genotype Depth < 10; Genotype Depth > 200
- For heterozygous calls: (A1 Depth + A2 Depth)/Total Depth < 0.9; A2 Depth/Total Depth < 0.2; Genotype likelihood[ref/ref] < 20; Genotype Depth < 10; Genotype Depth > 200
- For homozygous alternative calls: (A1 Depth + A2 Depth)/Total Depth < 0.9; A2 Depth/Total Depth < 0.9; Genotype likelihood[ref/ref] < 20; Genotype Depth < 10; Genotype Depth > 20; Genotype Depth > 200

Variant quality control

We then performed the variant-level quality control. We removed variants that failed the following filters:

- Call rate of < 90%

- Failed a liberal Hardy-Weinberg Equilibrium test (HWE) at $P < 1\times10^{-15}$ among unrelated samples
- Monomorphic in the final dataset

Sample quality control

We computed a number of quality metrics to identify bad-quality or duplicated samples. We used the high-quality autosomal variants present in both WES and array datasets to compute per-sample heterozygote concordance rates between WES calls and genotyping array calls.

We inferred the genetic sex of each participant with the --*check-sex* option in PLINK, using the high-quality independent X-chromosomal markers. The high-quality independent X-chromosomal markers were selected by the several steps listed below:

- Excluded pseudo-autosomal regions (bp 2781479 – 155701383)
- Filtered a list of variants in approximate linkage equilibrium by using –indep-pairwise, with 200 kilobase window size, 100 step size, and unphased-hardcall-r^2 threshold of 0.05
- Excluded call rate of < 90%
- Failed a liberal Hardy-Weinberg Equilibrium test (HWE) at $P < 1\times10^{-6}$ among unrelated samples

Supplementary Method 2 Relationship inference, kinship matrix, and principal component analysis

We initially selected a set of high-quality independent autosomal variants, which was defined as:

- variants without linkage equilibrium (200 kilobase window size, 100 step size, and unphased-hardcall-r^2 threshold of 0.1)
- Call rate ≥ 90%
- A liberal Hardy-Weinberg Equilibrium test (HWE) at $P < 1\times10^{-6}$ among unrelated samples
- minor allele frequency > 1×10^{-4}

Then, we constructed a 'WES-vs-array independent autosomal variants subset' with MAF > 0.1%, missingness < 1%, and HWE $P > 1\times10^{-6}$ in both the WES dataset and in the genotyping array data provided by the UK Biobank (among participants who had both available). Two rounds of pruning were further performed by using --indep-pairwise 200 100 0.1 and --indep-pairwise 200 100 0.05. We further removed indels and ambiguous SNPs and performed two rounds of pruning.

We used the KING-robust algorithm to compute pairwise kinship estimates for all samples in the dataset (using the high-quality independent autosomal variants in WES-vs-array subset). We then retained all information on pairs estimated to be genetically related to one another at 3rd degree or closer (kinship coefficient ≥ 0.0884). We used this data to construct a sparse kinship matrix in which all relationships with kinship coefficient < 0.0884 were set to 0.

We defined an unrelated subset of the WES cohort, where no relationships with kinship coefficient ≥ 0.0884 remained, a threshold that excludes any individuals related at 2nd degree

or closer. To maximize the sample size of this unrelated subset, we first iteratively removed individuals related to multiple other individuals until none remained. We then removed one sample from each remaining pair at random.

Principle components analysis was performed by plink2 using the high-quality independent autosomal variants. The first 10 principal components were adjusted as covariates.

Supplementary Method 3 Genotype and imputation of GWAS

Genotype data (version 3) of all 487,409 participants were from the UKB cohort. All blood samples were genotyped using the UK BiLEVE array and the UK Biobank axiom array. Details of the array design, genotyping, quality control, and imputation are available in a previous publication(Bycroft et al., 2018). We further removed samples that were outliers for heterozygosity or missingness, samples with putative sex chromosome aneuploidy, samples with a mismatch between self-reported and genetically inferred sex, samples not included in the central kinship inference, and samples who had revoked their consent. Imputed variants with per variant missing rates 1%, minor allele frequency < 1%, INFO<0.8, and deviation from the Hardy–Weinberg equilibrium with $P < 1\times10^{-12}$ were excluded from the analysis. In addition, samples that were estimated to have Caucasian ancestry (Field ID: 22006) and have no more than second-degree relatives were included in the analysis. We performed Logistic regression for binary phenotypes and linear regression for continuous traits with the PLINK 2.0 (Purcell et al., 2007) software.

To identify common variant associations near the identified rare variant signals, we ran common variant association analyses in the genomic region 500KB downstream and upstream of the identified gene. We adjusted for age, sex, and the first ten principal components. We then clumped and threshold the results to identify independent index common variants within the region using the --clump function in PLINK, using cutoffs of $P<1\times10^{-5}$ and $r^2<0.01$. Gene-based rare variant association analyses were then rerun within individuals who had both exome sequencing and imputed data available, adding each of the clumped common variants to the model as fixed-effect covariates.

In pinpointing GWAS signals that converged with common WES variants, the conventional threshold of $P<5\times10^{-8}$ was employed. Clump analysis was bypassed, as the objective was not to discern independent GWAS alleles but to identify signals nearby independent common WES variants (post-clump), which potentially modulated by WES variants. Furthermore, for protein GWAS employed as exposures in MR analysis, clump analysis was omitted, as this step is integrated within the MR.

Reference

Bycroft, C., Freeman, C., Petkova, D., Band, G., Elliott, L.T., Sharp, K., Motyer, A., Vukcevic, D., Delaneau, O., and O'Connell, J. (2018). The UK Biobank resource with deep phenotyping and genomic data. Nature *562*, 203.

Jurgens, S.J., Choi, S.H., Morrill, V.N., Chaffin, M., Pirruccello, J.P., Halford, J.L., Weng, L.C., Nauffal, V., Roselli, C., Hall, A.W.*, et al.* (2022). Analysis of rare genetic variation underlying cardiometabolic diseases and traits among 200,000 individuals in the UK Biobank. Nat Genet *54*, 240-250.

Purcell, S., Neale, B., Todd-Brown, K., Thomas, L., Ferreira, M.A., Bender, D., Maller, J., Sklar, P., De Bakker, P.I., and Daly, M.J. (2007). PLINK: a tool set for whole-genome association and population-based linkage analyses. The American journal of human genetics *81*, 559-575.
