# Supplementary material for: Large-scale whole-exome sequencing analyses identified protein-coding variants associated with immune-mediated diseases in 350,770 adults
Source: Nat Commun. 2024 Jul 15;15:5924. doi: 10.1038/s41467-024-49782-0 (PMC11250857; doi:10.1038/s41467-024-49782-0)
Supplement: Supplementary file 3 — Description of Additional Supplementary Files [file 41467_2024_49782_MOESM3_ESM.pdf]

Description of Additional Supplementary Files

File Name: Supplementary Data 1
Description: Baseline population characteristics

File Name: Supplementary Data 2
Description: Exome-wide rare variant analysis for 40 IMDs with $P<2.5\times10-6$

File Name: Supplementary Data 3
Description: Conditional analysis of exome-wide rare variants

File Name: Supplementary Data 4
Description: Multi-ancestry exome-wide rare variant analysis ($P< 0.05$)

File Name: Supplementary Data 5
Description: Case-control enrichment of rare variants across consequence categories

File Name: Supplementary Data 6
Description: Exome-wide common variants associated with IMDs ($P < 5 \times 10-8$)

File Name: Supplementary Data 7
Description: Positional mapping with eQTL and Chromatin interaction mapping by FUMA of common variants

File Name: Supplementary Data 8
Description: Genes overlapped between ANNOVAR and FUMA mapping

File Name: Supplementary Data 9
Description: Multi-ancestory of exome-wide common variant analysis ($P < 0.05$)

File Name: Supplementary Data 10
Description: GWAS convergence for the exome-wide common variants ($P< 5 \times 10-8$)

File Name: Supplementary Data 11
Description: Rare variant-based burden heritability of IMDs

File Name: Supplementary Data 12
Description: Exome-wide rare variant genetic correlations between IMDs estimated using BHR

File Name: Supplementary Data 13
Description: PheWAS analysis of rare variants associated with other clinical diseases ($P\_FDR < 0.05$)

File Name: Supplementary Data 14
Description: PheWAS analysis of common variants associated with other clinical diseases (P_FDR < 0.05)

File Name: Supplementary Data 15
Description: Longitudinal associations between the identified genes and risk of IMDs (P_FDR < 0.05)

File Name: Supplementary Data 16
Description: The intergroup differences of protein expression between mutation carriers and non-carriers (P_FDR < 0.05)

File Name: Supplementary Data 17
Description: Mendelian randomization analysis detecting the causalities (P_FDR < 0.05)

File Name: Supplementary Data 18
Description: Amino-acid signals predicted by identified mutations

File Name: Supplementary Data 19
Description: Proteomic analysis of rare variants assciated with altered protein expression (P_FDR < 0.05)

File Name: Supplementary Data 20
Description: Proteomic analysis of common variants assciated with altered protein expression (P_FDR < 0.05)

File Name: Supplementary Data 21
Description: Druggability for the rare genes by querying the GeneCards

File Name: Supplementary Data 22
Description: Druggability for the common genes by querying the GeneCards

File Name: Supplementary Data 23
Description: Effects of the identified rare genes on biologiccal indicators (P_FDR < 0.05)

File Name: Supplementary Data 24
Description: Effects of the identified common genes on biologiccal indicators (P_FDR < 0.05)

File Name: Supplementary Data 25
Description: GoClusters and protein-protein interaction analysis

File Name: Supplementary Data 26
Description: The diagnosis codes for IMDs

File Name: Supplementary Data 27
Description: The diagnosis Codes for immune-mediated diseases in FinGenn

File Name: Supplementary Data 28
Description: Biological indicators used in PheWas analysis

File Name: Supplementary Data 29
Description: Pleitropy effects across clinical diagnosis
