# Supplementary material for: Large-scale whole-exome sequencing analyses identified protein-coding variants associated with immune-mediated diseases in 350,770 adults
Source: Nat Commun. 2024 Jul 15;15:5924. doi: 10.1038/s41467-024-49782-0 (PMC11250857; doi:10.1038/s41467-024-49782-0)
Supplement: Supplementary file 5 — Reporting Summary [file 41467_2024_49782_MOESM5_ESM.pdf]

nature portfolio

Corresponding author(s): Jin-Tai Yu

Last updated by author(s): May 24, 2024

# Reporting Summary

Nature Portfolio wishes to improve the reproducibility of the work that we publish. This form provides structure for consistency and transparency in reporting. For further information on Nature Portfolio policies, see our Editorial Policies and the Editorial Policy Checklist.

## Statistics

For all statistical analyses, confirm that the following items are present in the figure legend, table legend, main text, or Methods section.

| n/a | Confirmed | |
|---|---|---|
| ☐ | ☒ | The exact sample size (*n*) for each experimental group/condition, given as a discrete number and unit of measurement |
| ☐ | ☒ | A statement on whether measurements were taken from distinct samples or whether the same sample was measured repeatedly |
| ☐ | ☒ | The statistical test(s) used AND whether they are one- or two-sided<br>*Only common tests should be described solely by name; describe more complex techniques in the Methods section.* |
| ☐ | ☒ | A description of all covariates tested |
| ☐ | ☒ | A description of any assumptions or corrections, such as tests of normality and adjustment for multiple comparisons |
| ☐ | ☒ | A full description of the statistical parameters including central tendency (e.g. means) or other basic estimates (e.g. regression coefficient) AND variation (e.g. standard deviation) or associated estimates of uncertainty (e.g. confidence intervals) |
| ☐ | ☒ | For null hypothesis testing, the test statistic (e.g. *F*, *t*, *r*) with confidence intervals, effect sizes, degrees of freedom and *P* value noted<br>*Give P values as exact values whenever suitable.* |
| ☒ | ☐ | For Bayesian analysis, information on the choice of priors and Markov chain Monte Carlo settings |
| ☒ | ☐ | For hierarchical and complex designs, identification of the appropriate level for tests and full reporting of outcomes |
| ☐ | ☒ | Estimates of effect sizes (e.g. Cohen's *d*, Pearson's *r*), indicating how they were calculated |

*Our web collection on statistics for biologists contains articles on many of the points above.*

## Software and code

Policy information about availability of computer code

| | |
|---|---|
| Data collection | No software was involved in data collection (data used is all directly available from UK Biobank, as described in detail in the paper). |
| Data analysis | Open-source R package SAIGE-GENE+ v1.1.6.2 was used to running gene-based collapsing tests for rare variants and the code was available from the GitHub (https://github.com/saigegit/SAIGE). PLINK v1.9 (https://www.cog-genomics.org/plink/1.9/) and v2.0 (https://www.cog-genomics.org/plink/2.0/) was adopted to perform association tests for common variants, variant quality control, and sample quality control. Hail is utilized to perform genotype quality control (https://hail.is/). KING 2.3.1(https://www.kingrelatedness.com/) is utilized to identify duplicated samples. Rare variants were annotated by SnpEff v5.1 (https://pcingola.github.io/SnpEff/se_introduction/) , ensembl variant effect predictor v101.0 (https://www.ensembl.org/info/docs/tools/vep/script/vep_download.html), and common variants were annotated by ANNOVAR (https://annovar.openbioinformatics.org/en/latest/). The burden heritability regression and genetic associations were performed using BHR v0.1.0 (https://github.com/ajaynadig/bhr) . Protein-protein interactions and pathway enrichment were performed by STRING V12.0 {https://cn.string-db.org/} and function annotations were performed by FUMA {https://fuma.ctglab.nl/}.<br>The code of the main analysis and visualization of single-nucleus RNA-seq data was an adaptation of the R package Seurat version 4.0 and available from https://satijalab.org/seurat/index.html. Mendelian randomization was performed by using the R package TwoSampleMR. The figures are generated using the 'ggplot2' and 'ggbreak' package. Codes are available at https://github.com/Sirius-Yang/IMDs_WES (DOI: 10.5281/zenodo.11307851). |

For manuscripts utilizing custom algorithms or software that are central to the research but not yet described in published literature, software must be made available to editors and reviewers. We strongly encourage code deposition in a community repository (e.g. GitHub). See the Nature Portfolio guidelines for submitting code & software for further information.

# Data

Policy information about availability of data

All manuscripts must include a data availability statement. This statement should provide the following information, where applicable:

- Accession codes, unique identifiers, or web links for publicly available datasets
- A description of any restrictions on data availability
- For clinical datasets or third party data, please ensure that the statement adheres to our policy

The analyses for this study were based on data from the UKB database (https://www.ukbiobank.ac.uk/) upon request under application number 19542. Data, analytic methods, and study materials are detailed above, and further details are available on supplementary materials. The gene-level and single variant association summary statistics generated in this study are provided in the Source Data file. For further information on study design, please refer to the Nature Research Reporting Summary linked to this article.

# Research involving human participants, their data, or biological material

Policy information about studies with human participants or human data. See also policy information about sex, gender (identity/presentation), and sexual orientation and race, ethnicity and racism.

| | |
|---|---|
| Reporting on sex and gender | We took sex into considerations in our study. Sex in the UK Biobank was determined based on self-reporting data via questionnaire, and all included participants gave written informed consent for sharing of individual-level data. |
| Reporting on race, ethnicity, or other socially relevant groupings | Ethnic background (Field ID 21000) and genetic ethnic grouping (Field ID 22006) was used to define ethnic backgrounds in this study. We included the White British samples with European descent genetic ethnic grouping in the main analysis and also conducted ancestry-specific analysis to investigate the generalisability of our findings. |
| Population characteristics | We analyzed a total of 350,770 European-descent individuals (mean age, 56.9 years; female sex, 162,210 [46.2%]) from the UKB for whom both WES data and phenotype data for IMDs were available (Supplementary Table 1). |
| Recruitment | The UKB is a population-based study that enrolled more than 500 thousand participants aged 40 to 69 years at recruitment across the UK. In-depth phenotypic, health-related, genetic and proteomic data were collected from a baseline assessment and subsequent follow-up visits. |
| Ethics oversight | UKB has obtained ethics approval from the Research Ethics Committee (REC; approval number: 06/MRE08/65) and informed consent from all participants. For this study, we included a total of 350,700 participants with available WES and clinical data after quality control (QC) under the project application number 19542. |

Note that full information on the approval of the study protocol must also be provided in the manuscript.

# Field-specific reporting

Please select the one below that is the best fit for your research. If you are not sure, read the appropriate sections before making your selection.

☒ Life sciences ☐ Behavioural & social sciences ☐ Ecological, evolutionary & environmental sciences

For a reference copy of the document with all sections, see nature.com/documents/nr-reporting-summary-flat.pdf

# Life sciences study design

All studies must disclose on these points even when the disclosure is negative.

| | |
|---|---|
| Sample size | We included a total of 350,700 participants with available WES and clinical data after quality control (QC). No statistical methods were used to predetermine sample sizes. |
| Data exclusions | Participants without whole-exome sequencing data, those failed to pass quality control (QC) were excluded.<br>In addition to the standard QC that was performed centrally, we performed additional genotype-, variant- and sample-based QC procedures to ensure a high-quality dataset for analyses49 (Supplementary Methods). In brief, we first conducted a genotype refinement on the preliminary genotype calls present within the pVCF files utilizing Hail. Multi-allelic sites were segregated to yield distinct bi-allelic representations. Any calls failing to meet the hard filtering criteria were removed. Then we performed variant-based QC by excluding variants that exhibited a call rate of less than 90%, deviated from Hardy–Weinberg equilibrium (P<1×10−15), or were present within regions of low-complexity. Finally, on the sample-level, we excluded participants who had rescinded their consent, instances of sample duplications, incongruences between genetically inferred sex and self-reported gender, as well as those samples displaying values for Ti/Tv, Het/Hom, SNV/indel, and singleton counts that deviated from mean±8 standard deviations. |
| Replication | In order to validate the gene-based associations in UKB, we searched from Kurki et al' s summary statistics analyzed from FinGenn dataset23 (Methods). Of the 35 disease phenotypes identified in the UKB, 24 were available in FinGenn, which covered 69 of the 92 identified genes. Searches yielded 13 associations (19% replicated) of Bonferroni-corrected significance (P<1.43×10-3; Supplementary Table 2). |
| Randomization | All association analyses incorporated covariates including sex, age, and the first 10 PCs as fixed effects, serving to attenuate confounders and |

| | |
|---|---|
| Randomization | mitigate potential population stratification. |
| Blinding | Blinding was not applicable to this study as this study is observational. |

# Reporting for specific materials, systems and methods

We require information from authors about some types of materials, experimental systems and methods used in many studies. Here, indicate whether each material, system or method listed is relevant to your study. If you are not sure if a list item applies to your research, read the appropriate section before selecting a response.

## Materials & experimental systems

| n/a | Involved in the study |
|---|---|
| ☒ | ☐ Antibodies |
| ☒ | ☐ Eukaryotic cell lines |
| ☒ | ☐ Palaeontology and archaeology |
| ☒ | ☐ Animals and other organisms |
| ☒ | ☐ Clinical data |
| ☒ | ☐ Dual use research of concern |
| ☒ | ☐ Plants |

## Methods

| n/a | Involved in the study |
|---|---|
| ☒ | ☐ ChIP-seq |
| ☒ | ☐ Flow cytometry |
| ☒ | ☐ MRI-based neuroimaging |

## Plants

| | |
|---|---|
| Seed stocks | *Report on the source of all seed stocks or other plant material used. If applicable, state the seed stock centre and catalogue number. If plant specimens were collected from the field, describe the collection location, date and sampling procedures.* |
| Novel plant genotypes | *Describe the methods by which all novel plant genotypes were produced. This includes those generated by transgenic approaches, gene editing, chemical/radiation-based mutagenesis and hybridization. For transgenic lines, describe the transformation method, the number of independent lines analyzed and the generation upon which experiments were performed. For gene-edited lines, describe the editor used, the endogenous sequence targeted for editing, the targeting guide RNA sequence (if applicable) and how the editor was applied.* |
| Authentication | *Describe any authentication procedures for each seed stock used or novel genotype generated. Describe any experiments used to assess the effect of a mutation and, where applicable, how potential secondary effects (e.g. second site T-DNA insertions, mosiacism, off-target gene editing) were examined.* |
